# Frontier interfacial water properties characterization and applications

**DOI:** 10.1093/nsr/nwaf284

**Published:** 2025-07-23

**Authors:** Ying-Ru Qiu, Wei Peng, Runze Zhang, Yao-Hui Wang, Wanlin Guo, Jian-Feng Li

**Affiliations:** College of Materials, State Key Laboratory of Physical Chemistry of Solid Surfaces, iChEM, College of Chemistry and Chemical Engineering, College of Energy, School of Life Sciences, College of Physical Science and Technology, and Discipline of Intelligent Instrument and Equipment, Xiamen University, Xiamen 361005, China; National Key Laboratory of Marine Corrosion and Protection, Luoyang Ship Material Research Institute, Xiamen 361100, China; College of Materials, State Key Laboratory of Physical Chemistry of Solid Surfaces, iChEM, College of Chemistry and Chemical Engineering, College of Energy, School of Life Sciences, College of Physical Science and Technology, and Discipline of Intelligent Instrument and Equipment, Xiamen University, Xiamen 361005, China; College of Materials, State Key Laboratory of Physical Chemistry of Solid Surfaces, iChEM, College of Chemistry and Chemical Engineering, College of Energy, School of Life Sciences, College of Physical Science and Technology, and Discipline of Intelligent Instrument and Equipment, Xiamen University, Xiamen 361005, China; Key Laboratory for Intelligent Nano Materials and Devices of Ministry of Education, State Key Laboratory of Mechanics and Control of Mechanical Structures, and Institute for Frontier Science, Nanjing University of Aeronautics and Astronautics, Nanjing 210016, China; College of Materials, State Key Laboratory of Physical Chemistry of Solid Surfaces, iChEM, College of Chemistry and Chemical Engineering, College of Energy, School of Life Sciences, College of Physical Science and Technology, and Discipline of Intelligent Instrument and Equipment, Xiamen University, Xiamen 361005, China; Innovation Laboratory for Sciences and Technologies of Energy Materials of Fujian Province (IKKEM), Xiamen 361005, China; College of Chemistry, Chemical Engineering and Environment, Minnan Normal University, Zhangzhou 363000, China; Liaoning Binhai Laboratory, Dalian 115004, China

**Keywords:** interfacial water, properties, characterization techniques, hydrovoltaic effect

## Abstract

Interfacial water is a unique form of water existing at the phase boundary. Due to its distinctive physical and chemical properties, it is significantly different from the ordinary bulk water and plays a crucial role in various scientific and technological fields. A deep understanding of interfacial water is expected to bring revolutionary applications in fields ranging from energy conversion to bionic intelligence. In this review, we start with clarification of the fundamental differences between interfacial water and bulk water, and comprehensively examine the structures, electrical properties, and hydrodynamics of interfacial water. Then, the principles and functions of advanced characterization techniques in revealing the moleculer-scale behavior of interfacial water are discussed. Experimental and computational advances in interfacial water research are reviewed to elucidate the key role of interfacial water in catalysis, energy storage, biological processes, and hydropower technologies, with the interaction between mechanistic insights and practical breakthroughs being emphasized. We further outline the prospects for advancing basic science and transformative applications, positioning interfacial water research as the cornerstone of the next generation of innovation.

## Teaser

This review elucidates the fundamental differences between interfacial and bulk water, summarizes the key properties and characterization techniques of interfacial water, highlights research advances across diverse applications, and outlines future directions.

## INTRODUCTION

Water is the foundation for the origin, survival, and evolution of life, and has an intricate connection with it. The unique properties of water make it an indispensable element in human activities and daily life. Over the past few centuries, significant progress has been made in understanding the structure of water, its phase transitions, and the characteristics of its hydrogen bonds. In nature, most water molecules exist in a tetrahedral coordination structure and are bound together through hydrogen bond interactions. However, in both scientific research and practical applications, interfacial water plays a crucial role.

Interfacial water is ubiquitously present at liquid-liquid, liquid-gas, solid-gas, and solid-liquid interfaces, and its structural and dynamic properties are strongly influenced by the nature of that interface. Based on the physicochemical properties of different interfaces, the structure and behavior of interfacial water show obvious system dependence, which can be broadly classified into five categories: (1) at air-water interfaces, water molecules form ordered hydrogen-bond networks that modulate surface tension and wettability [1,2]; (2) at water-organic solvent interfaces, the polarity of the solvent governs the configuration and mobility of interfacial water molecules [3]; (3) at oxide and mineral surfaces, surface functional groups and ionic sites direct the orientation and hydrogen bond structure of adsorbed water [4,5]; (4) at metal and alloy interfaces, the electronic structure of the substrate alters the polarization and spatial arrangement of nearby water layers [6]; (5) at biological interfaces, water exhibits highly ordered and spatially confined behavior near lipid membranes and protein surfaces [7,8]. However, in practical scenarios, the coexistence of multiple interfaces often gives rise to complex

interfacial phenomena. To disentangle these effects, this review mainly focuses on solid-liquid and liquid-liquid interfaces as representative systems with clearer interfacial characteristics. In such environments, structural modulation and hydrogen-bond reorganization of water molecules can significantly influence catalytic activity and selectivity, thereby enhancing material performance.

**Figure 1.**
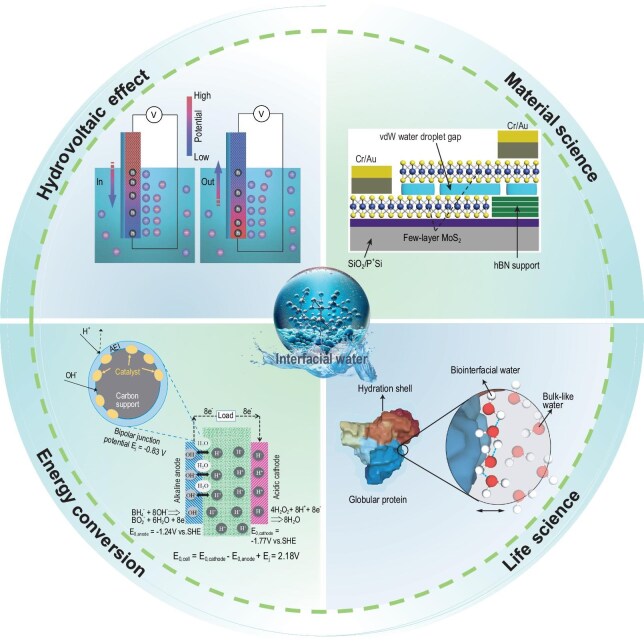
Schematic of the research and applications of interfacial water in various fields.

Interfacial water adheres to phase interfaces via intermolecular forces, including van der Waals forces, electrostatic interactions, and hydrogen bonds with fnctional groups or surface molecules. Due to its unique microscopic structure and physicochemical properties, interfacial water shows behaviors and features that are strikingly different from bulk water. Therefore, the investigation of interfacial water is essential for attaining a profound understanding and exercising effective regulation over the behavior of water molecules. Research on interfacial water traverses both fundamental and applied scientific arenas, including surface science, materials science, life science, hydrovoltaic technology, and energy conversion (Fig. 1). It assumes a pivotal and far-reaching role within these diverse disciplinary realms, underpinning advancements and enabling novel breakthroughs.

In the fields of materials and surface science, interfacial water holds the potential to engineer coating materials that exhibit exceptional anti-fouling or anti-icing properties by manipulating the distribution and state of water molecules on the surfaces of metals or polymers [9]. The adsorption and arrangement of interfacial water molecules on the surface of two-dimensional materials such as graphene and MoS₂ significantly affect the materials’ wettability, interfacial charge distribution, and lattice stress state, thereby exerting a significant influence on their electrical and mechanical properties [10,11]. Interfacial water influences the functional modulation, stability maintenance, and orchestration of biological processes associated with biomacromolecules in the field of life sciences. This is accomplished through its ability to regulate the fluidity of cell membranes, govern the complex process of protein folding, and modulate the hydrogen-bond network that underlies intermolecular interactions [12–14]. Furthermore, interfacial water directly participates in many important energy conversions and plays a significant role in regulating proton conduction, electrochemical reaction kinetics, and mass transfer processes. Particularly in fuel cells, water electrolysis, and CO₂ electroreduction systems [15–18], modulating the kinetic behavior of interfacial water can lower the activation energy barrier of electrochemical reactions, thereby enhancing catalytic efficiency and reducing costs. Moreover, hydrovoltaic effect is a phenomenon in which nanomaterials directly interacting with water to generate electricity without any additional inputs, which is an emerging green energy technology that occurs at the solid-liquid-gas triple-phase interface. At the interface between hydrovoltaic materials and interfacial water, a spontaneously formed electric double layer (EDL) consisting of a Stern layer and a diffusion layer owing to coulomb interactions. External forces (e.g. pressure, gravity) applied to interfacial water induce relative motion of the diffusion layer with respect to the Stern layer, and different electrokinetic phenomena will be produced. Among them, the streaming potential can convert mechanical energy into electric energy, providing a form of primary water energy harvesting based on direct solid-liquid interaction. Yin *et al*. accordingly discovered two new electrokinetic effects: drawing potential and waving potential [19,20], which were rated by international peers as ‘expanding the classic electrodynamics theory of two hundred years’. This has opened a new energy-harvesting era—hydrovoltaics [21].

However, due to the complexity of the interfacial structure and the highly dynamic properties of interfacial water, investigating its behavioral mechanisms presents a significant challenge. In recent years, with the advancements in characterization techniques, such as vibrational spectroscopy (including Raman, infrared, and sum-frequency), scanning probe microscopy, and X-ray absorption spectroscopy, combined with the development of theoretical calculation methods, researchers have not only revealed the dynamic behaviors of interfacial water at the macroscopic scale, but also elucidated its microscopic interaction mechanisms at the molecular level. Consequently, the research on interfacial water has considerably broadened our understanding of the behavior of water molecules across various processes. Its findings have not only propelled the advanced development of interfacial science but also, through the modulation of the dynamic behaviors of interfacial water, established a theoretical foundation and experimental paradigm for technological innovations in the fields of energy conversion and biology.

This review provides a comprehensive overview of several key aspects of interfacial water, including its definition, properties, and characterization techniques and applications. It begins by clarifying the fundamental definition of interfacial water and its distinction from bulk water. Then, the review turns to the physicochemical properties of interfacial water, including electrical properties and hydrodynamic behaviors. Recent advances in characterization techniques are subsequently reviewed, along with a discussion of its potential applications in electrocatalysis, energy storage, biology process, and hydrovoltaic technology. Finally, due to the structure and dynamic behavior of interfacial water itself being complex and the lack of a unified theoretical framework, future research directions for interfacial water are proposed to promote its development.

## FUNDAMENTAL OF INTERFACIAL WATER

### Differences and interplay between interfacial and bulk water

Bulk water refers to the macroscopic liquid region far from the interface, where intermolecular interactions between water molecules dominate. It typically forms a dynamic, disordered tetrahedral hydrogen-bond network with high molecular mobility. Bulk water exhibits a high dielectric constant (∼80 at 25°C), and its phase transitions follow classical thermodynamic principles. Another, interfacial water refers to the layer of water molecules that exists at the contact interface between water and other substances, typically on the nanometer level. On the molecular scale, within approximately two to three layers adjacent to the interface (e.g. electrode surfaces), water molecules exhibit a topological structure that is distinct from that of bulk water. This phenomenon occurs because when water molecules are in close enough proximity to the surface, the effects of the surface structure, surface chemical properties, and force field of the interface on water molecules are significantly different from those experienced in bulk water. Specifically, at the solid-liquid interface, various intermolecular forces (including surface tension, Coulombic interactions, and hydrogen bonds) significantly influence the properties of water molecules. These interactions can alter key properties such as molecular orientation, hydrogen bond network, and dielectric function, causing them to have different physicochemical properties compared to those of bulk water. Therefore, the unique physical and chemical environment at interfaces leads to interfacial water exhibiting distinct structural, dynamical, and electrical properties.

**Figure 2.**
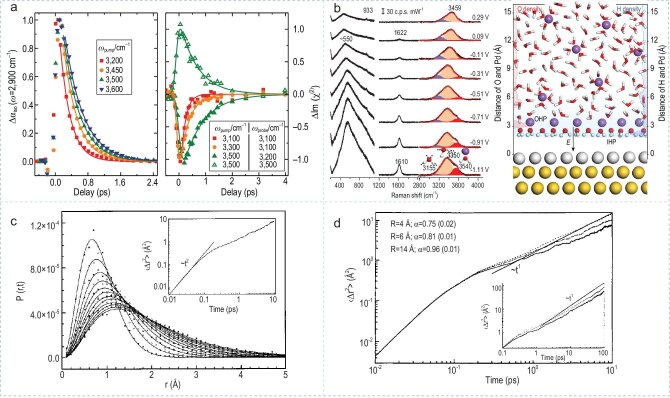
(a) Vibrational dynamics at OH stretching frequencies of bulk and interfacial H_2_O. Adapted with permission from [22]. Copyright 2015, Springer Nature. (b) Schematic of the interfacial model of the Pd/Au surface (right) and *in situ* Raman spectra of interfacial water (left). Adapted with permission from [23]. Copyright 2021, Springer Nature. (c) The probability distribution *P*(r, t) of water molecules within 4 Å from the protein surface as a function of time, and the relationship between the mean square displacement of water molecules and the free flight trajectory. Adapted with permission from [24]. Copyright 1998, American Physical Society. (d) Mean square displacements of water molecules versus time around a fully hydrated plastocyanin. Adapted with permission from [27]. Copyright 2002, American Chemical Society.

Interfacial effects markedly alter the structural, dynamic, and thermodynamic behaviors of water molecules, resulting in properties of interfacial water—such as O-H stretching vibrations, diffusion, and phase transitions that differ significantly from those of bulk water. These distinctions reflect the unique molecular configurations and hydrogen bond networks present at interfaces. For instance, Sietse *et al*. systematically revealed significant differences in the ultrafast vibrational relaxation dynamics between bulk and interfacial water by using time-resolved spectroscopy technologies. The strong dependence of the O-H stretching vibration frequency of interfacial water on the vibrational relaxation time reflects the significant regulatory effect of the interfacial environment on the hydrogen bond network structure of water molecules, leading to obvious structural heterogeneity of the interfacial water. (Fig. 2a) [22]. Wang *et al*. utilized electrochemical, *in situ* Raman spectroscopy, and computational techniques to investigate the configuration and dynamic evolution process of interfacial water molecules at the palladium single-crystal electrode/solution interface. This research described how interfacial water molecules gradually transition from an initial random distribution to an ordered arrangement at the molecular scale, reflecting the high responsiveness and tunability of the interfacial water molecule structure, and provided a crucial molecular-level perspective for exploring the role of interfacial water molecules in electrochemical reactions (Fig. 2b) [23].

Compared to the highly active motion behavior of bulk water molecules, interfacial water exhibits significant kinetic limitations under the combined influence of spatial confinement and interfacial interactions. Rocchi *et al*. conducted simulations of water molecule diffusion on protein surfaces and found that its motion no longer follows the Gaussian distribution predicted by Brownian motion. Instead, the mean squared displacement exhibits sublinear growth over time (Fig. 2c) [24]. This result suggests that the diffusion process of interfacial water is strongly regulated by its surrounding environment and exhibits non-classical diffusion characteristics. It highlights the necessity of incorporating non-equilibrium statistical mechanics or constrained diffusion theory when analyzing the migration and reactive behavior of interfacial water, in order to complement the limitations of traditional liquid water models.

In terms of thermodynamic properties, interfacial water also demonstrates phase transition behaviors that are fundamentally different from those of bulk water. Gragson and Richmond pointed out that the interfacial effect, by regulating the interactions between water molecules, significantly alters their hydrogen bond structure and energy distribution. When the surface potential changes, the interfacial water molecules undergo structural and hydrogen bond network reconstruction, leading to molecular arrangements and phase behaviors distinct from those of bulk water [25]. This finding not only deepens our understanding of the thermodynamic properties of interfacial water but also provides theoretical support for optimizing electrode–electrolyte interface processes and enhancing catalytic and energy conversion efficiencies.

However, interfacial water and bulk water are not independent of each other; instead, they form a coupled system through the interfacial region and jointly determine the physicochemical behavior of the interface. Interfacial water, constrained by spatial confinement and dominated by surface potential fields, exhibits ordered molecular arrangements, restructured hydrogen bond networks, and significantly slowed dynamics. These features directly influence key interfacial processes such as adsorption, wetting, and surface diffusion. Bulk water, on the other hand, supplies continuous energy and mass to the interface through heat conduction, flow, and material exchange, and can also exert feedback regulation on the interfacial state. This bidirectional coupling means that changes at the interface can propagate into the bulk phase and potentially influence the evolution of the entire system by inducing macroscopic changes in material properties.

Collins and Washabaugh proposed the Hofmeister effect, indicating that solutes determine the behavior of the adjacent first interfacial water layer, while the bulk solution determines the behavior of the third interfacial water layer, demonstrating the existence of a competitive and transformative relationship between interfacial water and bulk water [26]. Bizzarri and Cannistraro investigated the properties of protein hydration water through molecular dynamics simulations, comparing various structural and dynamic properties of interfacial water around proteins with those of bulk water. The findings revealed that both interfacial and bulk water significantly influence protein functionality. Interfacial water can influence the structure and dynamics of proteins through its interactions with the protein surface, thereby influencing protein function. In contrast, bulk water provides the solvent environment for proteins to perform biological functions effectively (Fig. 2d) [27].

Understanding the connection and differences between interfacial water and bulk water requires not only basing on existing theoretical models but also comprehensively considering the combined effects of multiple factors such as interfacial potential, surface chemical properties, and external environmental conditions. Current research has made certain progress in revealing the differences between the two. However, there are still many unanswered questions regarding how to precisely describe the dynamic behavior of interfacial water, changes in its hydrogen bond structure, and how these changes feed back into the thermodynamic properties of bulk water. In particular, how to quantify the interactions between interfacial water and bulk water and how to regulate these interactions in different application scenarios still require further exploration.

### Overview of solid-liquid and liquid-liquid interfacial water

**Figure 3.**
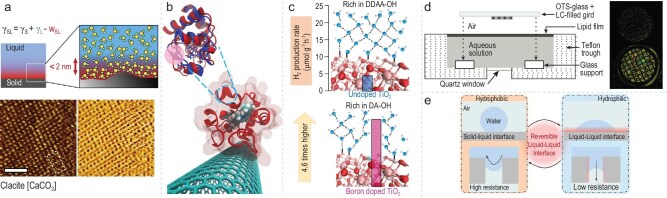
(a) Schematic representation of a solid-liquid interface at the molecular level and high-resolution images of single crystals of calcite in water. Adapted with permission from [29]. Copyright 2012, Copyright Clearance Center. (b) Snapshot of the most favorable adsorption conformation of superposition of the native Cyt c (blue) and adsorbed Cyt c (red) by interfacial water. Adapted with permission from [32]. Copyright 2019, American Chemical Society. (c) Schematic diagram of photocatalytic performance comparison between undoped TiO₂ and Boton doped TiO₂. Adapted with permission from [33]. Copyright 2024, American Chemical Society. (d) Configuration used for Langmuir–Schaefer transfer and polarized light micrographs of thin films of thermotropic liquid crystal 5CB for Langmuir–Schaefer transfer. Adapted with permission from [34]. Copyright 2008, American Chemical Society. (e) The liquid-liquid interface regulates the reversible wettability of surfaces and membranes between hydrophobic and hydrophilic states. Adapted with permission from [36]. Copyright 2022, American Chemical Society.

Solid-liquid interfacial water, which refers to the water present at the interface between the solid surface and the liquid, typically exhibits an ordered or partially ordered structure. The formation of ordered structure in interfacial water is influenced by both the chemical constitution and structure of the solid surface. Arsic *et al*. investigated the ordering properties of water adsorbed at room temperature on the surface of rock salt (100) under four different conditions. They discovered the existence of water monolayers with different ordering properties at the interface. Notably, the orderliness of the first layer of the water film increases with an increase in its thickness, and this ordering phenomenon at the solid-liquid interfacial water is closely related to solubility [28]. Voïtchovsky and Ricci and Ricci *et al*. elucidated the development of a water-ordered arrangement at the solid-liquid interface through atomic force microscopy and molecular dynamics simulations. The interfacial water at the solid-liquid boundary is influenced by the forces on the solid surface. On the surface of a charged solid, smaller water molecules can form a single hydration shell around charged atoms, thereby exhibiting an ordered structure (Fig. 3a) [29,30].

Furthermore, solid-liquid interfacial water plays a pivotal role in influencing material corrosion, biocompatibility, and catalytic reactions. The molecular structure and hydrogen bond network of interfacial water influence the activity of interfacial chemical reactions and the interaction between materials. In the corrosion process, when solid-liquid interfacial water contains corrosive ions (such as Cl⁻), it can act as a corrosive medium which interacts with the material surface through penetration and adsorption, directly affecting the rate of redox reactions and the formation of corrosion layers, thereby accelerating the material corrosion process. Hydrophobic coatings create an air layer by trapping a significant amount of air, which substantially reduces the actual contact area between solid-liquid interfacial water and the coating, effectively mitigating corrosion of materials [15,31]. The molecular arrangement and hydrogen bond network of the interfacial water directly influences the interaction between cells and the material surface as well as the degree of immune response [12,18]. Zhang *et al*. investigated the adsorption interactions between cytochrome *c* and carbon nanotube solutions from both dynamic and thermodynamic perspectives. They found that the thickness and structure of the interfacial water layer affect protein adsorption and cell adhesion (Fig. 3b) [32]. Verduci *et al*. combined FTIR with first-principles simulations to study two types of anatase TiO₂ (with/without B doping). They discovered that surface doping affects the local electric field, thereby influencing the response behavior of the hydrogen bond structure in the first layer of water molecules on the semiconductor surface, and modifying the reaction kinetics of water decomposition, revealing the role and properties of the first layer of water structure in contact with the photocatalyst surface. Furthermore, surface doping hampers the penetration of tetrahedrally coordinated water molecules into the sample and enhances defects within the hydrogen bond network, which provides a novel perspective on how solid-liquid interfacial water impacts catalytic reactions (Fig. 3c) [33]. This finding deepened the understanding of the structure-function relationship of the water layer on the catalyst surface.

Liquid-liquid interfacial water occurs between two liquids, as the boundary layer between two different liquid phases has its properties regulated by the intermolecular interactions between both liquids. Especially under the combined effects of interfacial tension and molecular arrangement between polar and nonpolar molecules, unique structural characteristics are exhibited. Liquid-liquid interfacial water not only promotes the formation of microphase separation and an emulsification phenomenon, it also regulates interfacial stability and mass transfer processes by constructing an interfacial membrane, thereby influencing multiphase reaction kinetics. Meli *et al*. proposed a new method of transferring Langmuir monolayers from the water surface to the oil-water interface using a metal grid. They found that the phase behavior of lipids at the liquid crystal-water interface is significantly different from that at the isotropic phase interface. This is because liquid crystals have a unique orientational order and can interact with lipids, affecting their phase behavior, and demonstrating the differences in the phase properties of different molecules at the liquid-liquid interface (Fig. 3d) [34]. Gao *et al*. utilized the liquid-liquid interface film formation method and developed a method for synthesizing highly crystalline and molecular sieving covalent organic framework (COF) films at an adjustable ionic liquid interface. Under the combined action of solvent viscosity and interfacial tension at the liquid-liquid interface, the COF films have high crystallinity, uniform and precisely controllable pore sizes, and exhibit high permeability and selective rejection of dyes [35]. Wu *et al*. introduced a liquid-liquid interface into the membrane system. The interfacial liquid transformed the solid-liquid interface into a liquid-liquid interface interaction, effectively enhancing water fluidity without affecting performance, indicating the important role of liquid-liquid interfacial water in both material synthesis and overall performance (Fig. 3e) [36].

## PHYSICOCHEMICAL PROPERTIES OF INTERFACIAL WATER

### Molecular structure of interfacial water and controlling factors

**Figure 4.**
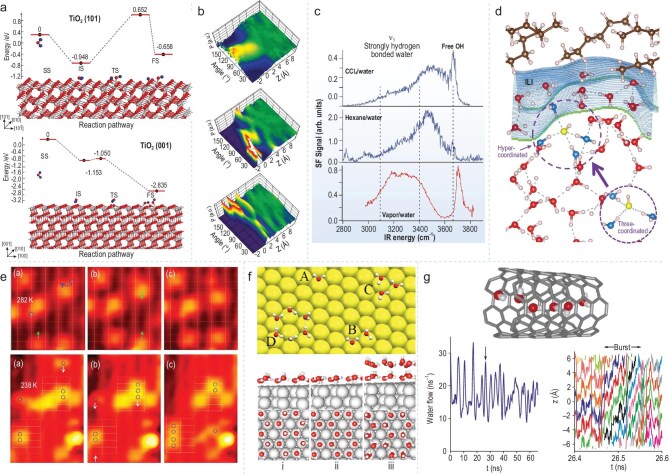
(a) Water adsorption conformations and decomposition reaction mechanisms on the anatase TiO₂ (101) surface vs. (001) surface. Adapted with permission from [38]. Copyright 2024, American Chemical Society. (b) Orientation distribution of water molecules as a function of their distance from the surface. Adapted with permission from [42]. Copyright 2009, American Chemical Society. (c) vibrational sum frequency (VSF) spectra of the CCl_4_/water, hexane/water, and vapor/water interfaces, indicating the differences in hydrogen bonds at the various interfaces. Adapted with permission from [43]. Copyright 2001, American Association for the Advancement of Science. (d) Snapshot illustration of H-bonding network of water with OH^−^ in the water-hexane interfacial region. Adapted with permission from [44]. Copyright 2020, American Physical Society. (e) A time-lapse sequence of high-magnification STM images (3.8 × 4.6 nm^2^) on RuO_2_ (110) exposed to H_2_O at 282 K and 238 K. Adapted with permission from [45] and [46]. Copyright 2015, American Chemical Society. Copyright 2014, American Chemical Society. (f) The water monomer and small clusters adsorbed on the Pt (111) surface and the H-up (i) H-down bilayers (ii) and the double-bilayers (iii) in the √3 × √3 *R* 30° symmetry on Pt (111). Adapted with permission from [48]. Copyright 2004, American Physical Society. (g) Structure of the hydrogen-bonded water chain inside, and flow of water through the nanotube. Adapted with permission from [51]. Copyright 2001, Springer Nature.

The adsorption behavior of water molecules and the dynamic reconstruction of the hydrogen bond network enable effective regulation of various interfacial phenomena, such as adsorption, wetting, and catalysis, by modulating the interfacial free energy states, surface tension, and chemical reactivity [37–39]. The adsorption of water molecules at the interface can be classified into physical adsorption and chemical adsorption. Senadheera *et al*. indirectly observed the process of water molecules condensing from water vapor and adsorbing on the solid surface using atomic force microscopy [40]. Zhao *et al*. utilized density functional theory calculations to investigate the adsorption behavior of water molecules on low-index anatase TiO₂ surfaces. By analyzing characteristics such as adsorption sites and molecular orientations, they revealed the distinct formation processes and differences of physical adsorption (water remains intact as a molecule) and chemical adsorption (water dissociates into OH⁻ and H⁺) of water molecules at the surfaces (Fig. 4a) [41].

At hydrophilic interfaces, water molecules establish a highly ordered and planar oriented dynamic hydrogen bond network through their electronegative oxygen atoms and partially positively charged hydrogen atoms. In contrast, at hydrophobic interfaces, the hydrogen bond network formed by water molecules appears loose and disordered. Wang *et al*. compared the orientational order of water molecules on talc and mica surfaces through molecular dynamics simulations, and found that the orientation and hydrogen bond configuration of water molecules on hydrophilic interfaces exhibit obvious orderliness (Fig. 4b) [42]. Scatena *et al*. probed hydrophobic interfaces, such as CCl₄/H₂O and hydrocarbon/H₂O, utilizing vibrational spectroscopy techniques. They found that the hydrogen bonds between adjacent water molecules at these interfaces were weaker than had been traditionally believed, contradicting the conventional model that predicts strong hydrogen bonds near fluid hydrophobic surfaces. This indicates that the hydrogen bond network of water molecules at hydrophobic interfaces exhibits a loose and disordered structure (Fig. 4c) [43]. Yang *et al*. designed a clean hydrophobic interface model and, through first-principles molecular dynamics simulations, found that water molecules form a disordered and loose hydrogen bond network on hydrophobic surfaces (Fig. 4d) [44]. These findings emphasized the importance of the hydrophilic/hydrophobic interface in regulating the hydrogen bond network structure of water molecules.

In interfacial science, temperature and pressure serve as crucial external variables that significantly influence the adsorption behavior, structure, and properties of interfacial water. This regulation occurs through alterations in the kinetic energy of water molecules and intermolecular interactions. Elevated temperatures increase the kinetic energy of water molecules, resulting in more vigorous thermal motion which weakens the stability of hydrogen bonds, subsequently decreasing the orderliness in the arrangement of interfacial water molecules, as well as adsorption strength. Conversely, an increase in pressure enhances the interactions among water molecules as well as between water molecules and the surface. This improvement leads to a higher degree of ordering in interfacial water molecules and increases the compactness of the hydrogen bond network. Mu *et al*. employed variable temperature scanning tunneling microscopy combined with theoretical calculations to observe the adsorption, diffusion, and cluster formation processes of water molecules on a coordinatively unsaturated ruthenium surface. When the temperature is ∼277 K, water dimers start to diffuse and exhibit a higher diffusion barrier than water monomers. Additionally, even-numbered water molecule clusters are stably present through strong internal hydrogen bonds, while odd-numbered water molecule clusters are relatively unstable under thermal equilibrium, indicating that temperature variation plays a crucial role in regulating the arrangement of water molecules at the interface and the reorganization of the hydrogen bond network (Fig. 4e) [45,46]. Lyapin *et al*. obtained the correlation between the density and ultrasonic velocity of H₂O I*h* ice through experiments. An increase in pressure promotes multiple phase transitions of H₂O I*h* ice, altering the interactions between water molecules. Water molecules form compact circular or helical networks through bent hydrogen bonds [47].

Furthermore, interfacial materials (including metals, oxides, and carbon-based) possess unique surface properties that can influence the arrangement of water molecules. Metal surfaces, characterized by their high electron density and unique surface states, can directly regulate the adsorption of water molecules through charge transfer mechanisms or the formation of metal-oxygen coordination bonds. Meng *et al*. analyzed in detail the adsorption behavior of water on different metal surfaces using density functional theory, and revealed that the interaction between water and metal surfaces is dominated by the lone pair-*d* band coupling through the surface states. This bonding mode is highly localized within the contact layer, meaning that the interaction between water molecules and the metal surface is local and highly spatially dependent, thereby affecting the adsorption structure and kinetic properties of water. Furthermore, phenomena such as the enhancement of hydrogen bonds in the adsorption structure on the metal surface and the state transition of H atoms were also observed (Fig. 4f) [48]. This result underscores the importance of the interaction between water and metal surfaces. The active sites on the oxide surface, such as hydroxyl groups and oxygen vacancies, not only promote the directional adsorption and dissociation of water molecules but also regulate the hydrogen bond reorganization and proton migration processes of interfacial water, thereby influencing the kinetics of interfacial reactions. Zhang *et al*. investigated the intermediates of water oxidation catalyzed by Co₃O₄ nanoparticles through time-resolved Fourier-transform infrared spectroscopy. The temporal behavior of the intermediates indicates that they belong to different catalytic sites, highlighting the interaction between interfacial water molecules and catalytic sites in the water oxidation reaction mechanism, and provides important experimental evidence for regulating interfacial water molecules through metal oxides [49].

Finally, carbon-based materials can regulate the behavior of water molecules by adjusting their surface functional groups and interlayer spacing. Kalra *et al*. investigated the permeation behavior of seawater through a hexagonal structured carbon nanomembrane under an osmotic gradient using molecular dynamics simulations. They found that the carbon membrane exhibits highly efficient regulation of water molecules. The water flow is almost friction free. The interlayer spacing of the membrane decreases after a short period of time and is affected by the structured water layer in the long term, with a water flow rate of 5.8 molecules per nanosecond. Furthermore, within this layer, water molecules establish a hydrogen bond network that necessitates significant external force to disrupt, which renders the water layer highly stable [50]. Hummer *et al*. discovered through molecular dynamics simulations that, at the nanoscale, water molecules within non-polar carbon nanotubes form one-dimensional ordered chains and exhibit pulsed transmission. Carbon nanotubes precisely regulate their interaction with interfacial water, enabling water molecules to form stable hydrogen bonds and relatively stable electric dipole moment orientations (Fig. 4g) [51].

### Electrical properties of interfacial water

**Figure 5.**
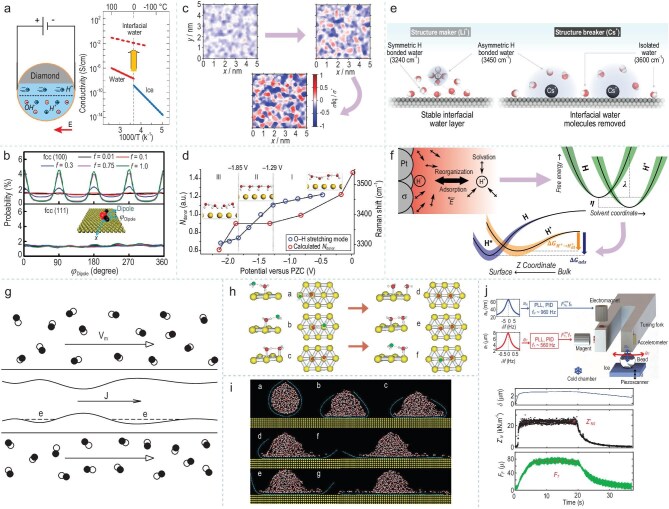
(a) Schematic diagram of radio frequency measurement of interfacial water on nanoporous diamond and DC conductivity σ as a function of temperature. Adapted with permission from [52]. Copyright 2020, American Chemical Society. (b) Ordered structure of interfacial water at the fcc (111) and fcc (100) hydrophilic surface, and contrasting changes in the order parameter η of interfacial water with respect to f on the fcc (100) and fcc (111) surfaces. Adapted with permission from [53]. Copyright 2021, American Chemical Society. (c) Role of water-water interactions in the response of water's interfacial molecular structure to surface charge. Adapted with permission from [54]. Copyright 2021, licensed under CC BY 4.0. (d) Schematic representation of an EDL model and potential-dependent evolution of the hydrogen bond network of interfacial water. Adapted with permission from [56]. Copyright 2019, Springer Nature. (e) A schematic showing that Li^+^ promotes symmetric H-bonded water at the interface while Cs^+^ tends to form isolated water molecules at the Au surface. Adapted with permission from [58]. Copyright 2024, Springer Nature. (f) Schematics of Volmer reaction along the collective solvent coordinate and proton surface distance, z. Adapted with permission from [59]. Copyright 2023, Springer Nature. (g) Scheme of a metallic nanotube immersed in a liquid, flowing along it with a bulk velocity *v*_m_. Adapted with permission from [60]. Copyright 2001, American Physical Society. (h) Mechanism for water dimer diffusion on Pd (111). Adapted with permission from [61]. Copyright 2004, American Physical Society. (i) The dynamic electro-elasto-capillary process of a droplet on gold substrate. Adapted with permission from [62]. Copyright 2010, American Physical Society. (j) Schematic of the setup of stroke-probe tribology of ice (up) and steady-state friction force *F_T_* as a function of tangential speed *U* (down). Adapted with permission from [64]. Copyright 2019, licensed under CC BY 4.0.

The electrical properties of interfacial water, including electrical conductivity, dielectric functions, and surface charge distribution, regulate the kinetics of chemical reactions by influencing the activation energy and reaction rate of interfacial reactions. First, the electrical conductivity of interfacial water refers to the ability of water molecules to migrate charges in the vicinity of a solid surface. Artemov *et al*. utilized radio-frequency technology to measure the electrical properties of interfacial water within the nanoporous matrix formed by diamond particles. They systematically determined the electrical conductivity of pure interfacial water for the first time, and the observed protonic conductivity was 5 orders of magnitude higher than that of bulk water, subverting the traditional understanding of the electrical conductivity of interfacial water (Fig. 5a) [52].

Second, the dielectric function of interfacial water reflects the degree of response of water molecules to an electric field, and its value is lower than that of bulk water. Qi *et al*. discovered that the parallel dielectric constant of water on the solid surface is determined by the solid-liquid interaction and the structure properties of interfacial water on the solid surface. Due to the specific antiferroelectric ordering of interfacial water molecules, the amplitude of dipole fluctuations associated with both the number of hydrogen bonds and the orderliness of interfacial water is curtailed, resulting in a significant decrease (∼44%) in the parallel dielectric constant of water molecules at the interface (Fig. 5b) [53]. Finally, the residual charge at the interface serves as the basis for influencing the directional arrangement of interfacial water molecules and the mechanism of electrostatic interactions. This provides crucial evidence for analyzing the electron transfer pathways during the charge transfer process, the reaction kinetics, and the formation and influence of the interfacial electric field. Shin and Willard introduced a new computational method to quantify the nonlinear polarization response of water, which can determine the effective surface charge distribution of hydrated surfaces over atomistic length scales (Fig. 5c) [54]. Uysal *et al*. found that water molecules on a hydrophobic surface form specific structures and orientations. Given that water molecules possess polar characteristics with dipole moments, variations in their arrangement and density result in corresponding changes in charge distribution [55].

In addition, the electric field and electrode potential serve crucial regulatory functions in determining the electrical properties of interfacial water, including polarization, dielectric constant, and ion migration. The electric field influences the polarization degree of interfacial water molecules by modifying their orientation and charge distribution. By modulating the electrode potential, the strength and characteristics of the electric field at the interface can be modified, which subsequently influences the dielectric constant of interfacial water molecules and their interaction with substances. The regulatory effects of these two factors determine ion migration behavior, subsequently influencing the efficiency of electrochemical reactions and the stability of the interface. Li *et al*. combined *in situ* Raman spectroscopy and *ab initio* molecular dynamics simulations, revealing the structural evolution process of interfacial water at the Au single-crystal electrode surface during the potential scanning process. Towards negative potentials, the interfacial water molecules evolve from a ‘parallel’ structure to ‘one-H-down’ and then to ‘two-H-down’. This finding, for the first time at both the experimental and theoretical levels, correlated the configuration transformation of interfacial water and the breaking of hydrogen bonds with a precise electrode potential (Fig. 5d) [56]. Xu *et al*. used sum-frequency spectroscopy to observe the structural evolution of water molecules at the graphene-water interface under the regulation of an electric field (Fig. 5e). The dangling O-H bonds disappear when the onset of the hydrogen evolution reaction occurs. This structural change is caused by the excess intermediate species accumulated next to the electrode, revealing the direct effect of electrode potential on the structure and electrical behavior of interfacial water molecules [57].

Meanwhile, Tian *et al*. regulated the electrode potential from the potential of zero charge to a negative potential, making the surface of the Au electrode negatively charged, which enhanced dipole interaction with water molecules. Meanwhile, they changed the states of different cations at the interface to affect the hydrogen bond network structure of water, thus altering the electrical properties of interfacial water [58]. Electrode–electrolyte interface will lead to the hardening of the hydrogen bond network of water molecules when strengthening the electric field, the slow orientation relaxation of water molecules at the interface decreased the dielectric constant. When the electrode potential becomes negative, the relaxation of interfacial water is accelerated, thereby influencing the dielectric constant. Concurrently, the electrostatic potential generated by the charge on the electrode surface altered the electrochemical potential of ions in the EDL, thus influencing the behavior of migrating ions. Wilson *et al*. used dynamics simulations to verify this phenomenon (Fig. 5f) [59].

### Hydrodynamic behaviors of interfacial water

The electrical properties of interfacial water arise from the uneven charge distribution caused by the polarity of their molecules, leading to the formation of an EDL structure with both positive and negative charges at solid-liquid interfaces. This structure endows the interfaces with unique electrical behaviors. When relative motion occurs between the EDL at solid-liquid interfaces, water driven by surface tension flows along the solid surface, carrying adjacent charges for directional movement. Král and Mo interpreted this phenomenon as friction-induced excitation of phonons in solid materials through water slippage along the surface, thereby driving directional charge transport and generating streaming potential phenomena [60]. The EDL influences the fluid's surface tension, viscosity, and slip properties through the coupling of interfacial charge and intermolecular forces.

Understanding the hydrodynamic behaviors of interfacial water is equally critical. Ranea *et al*. proposed a novel diffusion mechanism of water dimers on a metal surface, which relies on the quantum tunneling rearrangement ability of hydrogen bonds. The quantum tunneling effect and hydrogen bond rearrangement are conducive to the diffusion of water dimers, increasing the mobility of interfacial water molecules. Quasifree rotation and dynamic exchange enable the interfacial water to reduce the surface tension and exhibit abnormal fluidity (Fig. 5h) [61]. Yuan and Zhao utilized molecular dynamics simulations to explore the characteristics of the precursor film (PF) in dynamic wetting and electrowetting. They found that the solid like and no slip propagation behavior of the PF was attributed to the high viscosity of interfacial water. This high viscosity regulates the rate and distribution of water molecules moving along the interface, revealing its rapid propagation and the power-law relationship in terms of time (Fig. 5i) [62]. Zhu and Granick investigated ionic interfacial water confined between mica crystals with a thickness of 1–2 water molecules. They discovered that the effective shear viscosity and frequency-dependent dynamic oscillatory shear spectra of such water exhibit significant variations with twisting angles. The effective viscosity varied by several orders of magnitude as the twist angle was changed, demonstrating the significant influence of surface lattices on the viscosity of interfacial water. It provides a crucial foundation for understanding the hydrodynamic behavior of interfacial water in fields such as colloid suspension and soil science [63]. Canale *et al*. developed a stroke-probe force measurement technique to uncover nanorheology of interfacial water during ice gliding (Fig. 5j). They discovered that the interfacial water on ice exhibits complex viscoelastic rheological behavior, with a viscosity up to 2 orders of magnitude larger than pristine water. This finding not only provides a new explanation for the slipperiness of ice, but also indicates that the reduction in friction by hydrophobic coatings is associated with alterations in the viscosity of the interfacial water [64].

## CHARACTERIZATION TECHNIQUES OF INTERFACIAL WATER

Due to the complexity of interfacial structures and the highly dynamic nature of interfacial water, its behavior and underlying mechanisms have remained a focal point and challenge in scientific research [65–67]. In recent years, with the continuous emergence of advanced characterization techniques and the rapid development of theoretical computational methods, researchers have not only revealed the dynamic properties of interfacial water at the macroscopic level, but have also elucidated its microscopic behavior at the molecular and atomic levels. Currently, there is a wide range of experimental techniques available for characterizing the properties and structure of interfacial water. In this paper, we primarily discuss techniques based on vibrational spectroscopy (including Raman, infrared, and sum-frequency), X-ray absorption spectroscopy, and scanning probe microscopy (such as scanning tunneling microscopy and atomic force microscopy).

**Figure 6.**
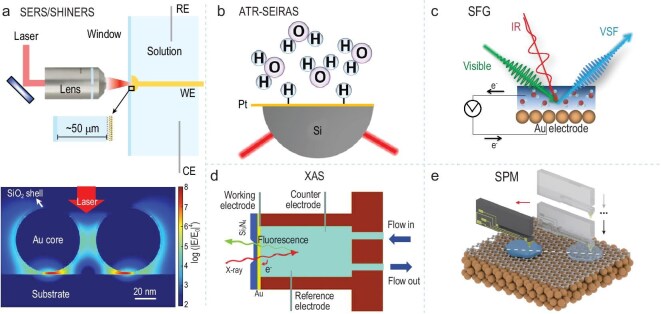
Characterization techniques for interfacial water. (a) SERS/SHINERS. Adapted with permission from [23] and [70]. Copyright 2021, Springer Nature. Copyright 2023, Springer Nature. (b) ATR-SEIRAS. Adapted with permission from [77]. Copyright 2020, American Chemical Society. (c) SFG. Adapted with permission from [81]. Copyright 2017, Wiley-VCH. (d) XAS. Adapted with permission from [88]. Copyright 2014, American Association for the Advancement of Science. (e) SPM. Adapted with permission from [89]. Copyright 2024, American Association for the Advancement of Science.

In practical research, interfacial water often coexists with bulk water. The signals from bulk water can obscure the properties of interfacial water, making it difficult to accurately characterize its properties. Researchers have used various strategies to reduce bulk water interference. Interface-selective techniques are preferred, such as second-harmonic generation (SHG) spectroscopy. These techniques are sensitive only to molecules at the interface, avoiding bulk water interference. For non–interface-selective techniques, like Raman and infrared spectroscopy, experimental setups can be designed to minimize bulk water interference. For example, in enhanced infrared spectroscopy, choosing the right measurement angle and optical path design can reduce the contribution of bulk water to the signal [68,69]. In enhanced Raman spectroscopy, plasmonic nanostructures can confine interfacial water to the gap between the substrate and nanoparticles (hotspot region). This also helps reduce the relative contribution from bulk water in the spectral analysis (Fig. 6a) [23,70].

To obtain deeper insight into the properties of interfacial water, researchers have employed a combination of experimental techniques, along with theoretical calculations such as molecular dynamics simulations and density functional theory (DFT) [71]. Theoretical calculations provide explanations for experimental phenomena at the atomic and molecular levels and predict the behavior of interfacial water, offering crucial theoretical guidance for experimental research. The integration of different methods allows each to leverage its strengths, validate each other's findings, and compensate for the limitations of a single approach. This section will introduce commonly used experimental techniques and their combinations, as well as theoretical methods. The focus will be on their innovations in eliminating bulk phase interference, providing a foundation for advancing the study of the functions and roles of interfacial water.

### Vibrational spectroscopy

Raman spectroscopy occupies a crucial position for studying interfacial water, providing insights into molecular structure and chemical bonding. It complements infrared spectroscopy, although its signals are typically weaker. The advent of surface-enhanced Raman spectroscopy (SERS) and shell-isolated nanoparticle-enhanced Raman spectroscopy (SHINERS) has significantly broadened the scope of Raman applications [72,73]. SERS, in particular, offers distinct advantages in interfacial water research. Due to the low number of interfacial water molecules, conventional Raman signals are weak and difficult to detect. SERS can greatly enhance Raman signals, with reported enhancement factors ranging from 10⁶ to 10¹⁴. However, the actual enhancement strongly depends on the material composition, morphology, and design of the metal nanostructures employed. The resolution of conventional SERS is limited by the optical diffraction limit, ∼500 nm, in the visible light wavelength range. When combined with tip-enhanced Raman scattering (TERS), it can be broken through to <10 nm (depending on the radius of curvature of the needle tip). However, the resolution and signal stability are significantly affected by laser focusing, substrate uniformity, and thermal effects. This technique selectively captures information from interfacial water, avoiding interference from bulk water, thus enabling the study of molecular vibration modes and hydrogen bond structures of interfacial water. However, the complex environment of interfacial water makes direct SERS measurements prone to interference from impurities.

SHINERS technology overcomes this limitation by coating the surface of metal nanoparticles with a chemically inert protective shell, such as SiO₂ or Al₂O₃. This shell isolates the metallic core from reactive impurities in complex environments and prevents unwanted interactions with interfacial water molecules. Meanwhile, it allows the enhanced electromagnetic field generated by the metallic core to penetrate the shell and interact with nearby molecules, thus preserving the Raman enhancement effect. This mechanism enables stable and reproducible analysis of the structure and dynamic behavior of interfacial water in complex systems. Li's group has been conducting *in situ* Raman spectroscopy studies of interfacial water for years. For instance, they combined *in situ* SERS with molecular dynamics simulations to monitor real-time changes of interfacial water at the Pd single-crystal electrode/solution interface during electrocatalytic hydrogen evolution. Their research revealed that the presence of interface cations promotes the ordering of water molecules, accelerating charge transfer and enhancing the electrocatalytic hydrogen evolution rate [23].

Infrared spectroscopy (IR) is also based on molecular vibration principles. It identifies molecular vibration modes by measuring the absorption of infrared light. IR spectroscopy is highly sensitive to variations in the hydrogen bond network of interfacial water, enabling the detection of subtle changes in hydrogen bond environments. This sensitivity provides insights into the interactions between interfacial water and surrounding molecules. Common modes of surface infrared spectroscopy include the external reflection mode (typically called IRAS or IRRAS) [74,75], and the internal reflection mode (attenuated total reflectance, known as ATR-IR or ATR-FTIR) [76]. Compared to regular IRAS, ATR-IR spectroscopy offers higher surface sensitivity and specificity. During *in situ* electrochemical monitoring, ATR-IR can achieve millisecond time resolution without the need for a thin solution layer. Furthermore, by incorporating surface-enhanced infrared techniques, ATR-SEIRAS spectroscopy's sensitivity increases by 10–10^6^ times, and detection limit can reach 10^−^^6^ to 10^−^^8^ M, which is suitable for interface science research. The conventional resolution of ATR-SEIRAS is at the micrometer level (3–20 μm), which is suitable for macroscopic analysis of the chemical composition of the bulk phase or interface. If nanometer-level resolution is required, it depends on near-field coupling technology. The behavior of interfacial water molecules and hydrogen atoms adsorbed on electrode surfaces can be studied by ATR-SEIRAS (Fig. 6b) [77] as well as how hydrogen bond networks affect catalytic performance. For example, using ATR-SEIRAS combined with molecular dynamics simulations, it was found that specific molecular additives, such as N-methylimidazole, could significantly improve the hydrogen evolution reaction/hydrogen oxidation reaction (HER/HOR) performance of Pt electrodes by modulating the hydrogen bond network of interfacial water [78]. This study provides both experimental and theoretical support for optimizing interfacial water regulation in catalytic applications.

SHG spectroscopy, as a nonlinear optical technique, offers surface and interface selectivity. It allows for the specific detection of the structure and dynamics of interfacial water without damaging the sample [43,79,80]. For instance, an *in situ* electrochemical setup was used to study the structure of water molecules at the Au electrode/solution interface (Fig. 6c). This technique eliminates the interference from bulk water molecules on infrared light absorption [81]. Additionally, by combining sum-frequency generation (SFG) and Raman spectroscopy, both experimental and theoretical approaches were used to research the behavior of deuterated water (D_2_O) on a graphene surface under an electric field. The study revealed that the behavior of interfacial water molecules in the applied electric field is not simply a linear dielectric response, which has significant implications for refining theoretical models [82].

### X-ray absorption spectroscopy (XAS)

X-ray absorption spectroscopy (XAS) is an element-specific technique that provides detailed information about the coordination environment around atoms [83]. XAS measurements are typically classified by energy range into soft X-rays (<2000 eV), medium-energy X-rays (2000–4000 eV), and hard X-rays (>4000 eV). Soft X-ray absorption spectroscopy (sXAS) is particularly suitable for detecting light elements (such as C, N, O, F) and transition metals’ 1s or 2p orbitals [84].

The detection modes of XAS include total fluorescence yield (TFY) and total electron yield (TEY). TFY is used for bulk phase measurements, while TEY is more suitable for surface analysis [85]. Since X-rays interact strongly with matter, a vacuum environment is typically required for XAS measurements. To overcome the vacuum limitation, X-ray-transparent films have been used to encapsulate liquid samples in both sXAS and transmission electron microscopy applications [86,87]. For example, a study designed a special electrochemical cell that used a Si_3_N_4_ membrane to separate the vacuum environment of the sXAS chamber from the liquid electrolyte in a conventional setup (Fig. 6d) [88]. This study investigated the structure of interfacial water on a gold electrode surface. The researchers found that the structure of interfacial water differs significantly from bulk water and is influenced by the surface potential of the gold electrode.

### Scanning probe microscopy

Scanning probe microscopy (SPM) techniques (atomic force microscopy [AFM], scanning tunneling microscopy [STM]) allow direct observation of the microstructure and behavior of interfacial water at the nanoscale. These techniques provide a robust apparatus for exploring the local properties of interfacial water. For example, Fig. 5e illustrates the manipulation of two-dimensional ice sliding with a tip, showcasing the application of SPM in studying the dynamic processes of interfacial water (Fig. 6e) [89]. Jiang's group has made significant contributions in this field. They used ultra-high-resolution AFM to reveal the atomic structure of hydrated ions and to understand corrosion resistance mechanisms at solid-liquid interfaces. These techniques have now been extended to practical, cross-disciplinary issues such as energy, surface anti-icing, and electrochemical water splitting for hydrogen production. This expansion provides new perspectives on the role of interfacial water in various applications [90].

## APPLICATIONS OF INTERFACIAL WATER

### Electrocatalysis and energy storage

**Figure 7.**
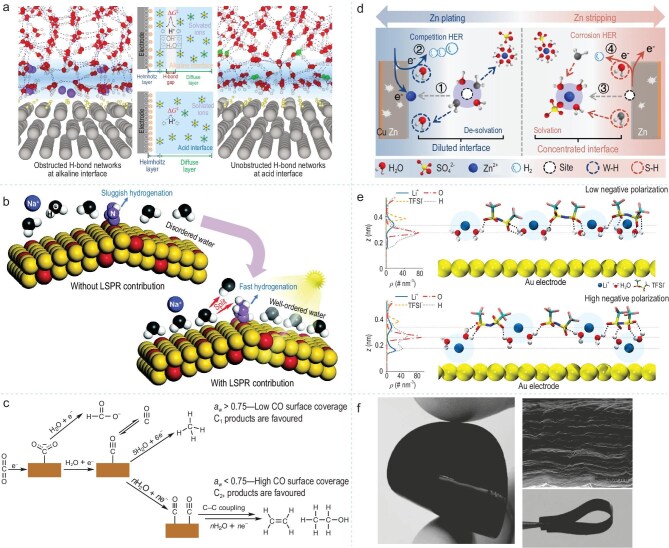
(a) Schematic diagram of the acid and alkaline EDL structure and the proton transfer from bulk to interface across the H-bond gap. Adapted with permission from [91]. Copyright 2022, Springer Nature. (b) Schematic of water moleculer behavior in the NRR process without (up) and with (down) light illumination, indicating plasmon-triggered ordering of interfacial water molecules. Adapted with permission from [92]. Copyright 2024, Wiley-VCH. (c) Schematic depicting differences in the mechanism of CO_2_ reduction on Cu electrodes for low and high *a*_w_. Adapted with permission from [93]. Copyright 2023, Springer Nature. (d) Schematic diagram of the relationship between hydrogen bond evolution and HER during the zinc plating/stripping process. Adapted with permission from [94]. Copyright 2024, American Chemical Society. (e) Schematic atomistic EDL structure in highly concentrated aqueous electrolyte under low negative polarization, from 0 to −0.6 V. Adapted with permission from [95]. Copyright 2022, Springer Nature. (f) Photographs of the as-formed flexible self-stacked, solvated graphene (SSG) films and SEM image of the cross-section of a freeze-dried SSG film (upper right). Adapted with permission from [96]. Copyright 2011, Wiley-VCH.

Interfacial water, through dynamic restructuring of the hydrogen bond network and the regulation of orientation, synergistically interacts with electric field intensity and surface-adsorbed species to modulate the adsorption energy barriers of reaction intermediates as well as proton transfer efficiency, thus optimizing the electron transfer pathways and interfacial charge transport, which in turn influences the reaction kinetics of catalytic active centers, the product distribution, and the efficiency of the catalytic process. Li *et al*. investigated the reaction kinetics of the HER that occurs on the Pt (111) electrode under acidic and alkaline conditions. They found that the interconnectedness of its hydrogen bond network and the distribution of water in the EDL play a dominant role in the HER process. The adsorbed OH⁻ optimizes the HER performance by regulating the connectivity of interfacial water and the architecture of its hydrogen bond network, providing a new strategy for the development of pH regulated catalysts (Fig. 7a) [91]. Zhang *et al*. constructed AuCu pentagonal nanoparticles as plasmonic catalysts and verified at the molecular scale that the strong local electric field induced by the plasmonic effect can significantly enhance the degree of order in the arrangement of interfacial water, thereby promoting interfacial electron transfer and proton generation. This enables the catalyst to exhibit excellent nitrogen reduction reaction performance (Fig. 7b) [92]. Zhang *et al*. studied the influence of H_2_O activity on the electrochemical reduction of CO₂ to form multi-carbon products on a copper electrode. They found that the high-concentration electrolyte can disrupt the hydrogen bonds of interfacial water and reduce H_2_O activity, causing most water molecules to coordinate with ion pairs such as Na⁺. This reveals the important role of interfacial water in controlling the branching between the C_1_ and C_2+_ pathways during CO₂ reduction (Fig. 7c) [93].

Furthermore, by virtue of its unique properties of hydrogen bond network evolution and ion transport, interfacial water collaborates with the cation solvation effect to optimize the structure of the EDL at the electrolyte/electrode–electrolyte interface. It can significantly enhance ion transport efficiency, charge storage capacity, and power density. Yu *et al*. discovered that during the electroplating/stripping process of metallic zinc in a ZnSO₄ electrolyte, the enrichment of interfacial water and the evolution of its hydrogen bond network directly affect the generation of H₂ and the overall efficiency of the battery. The solvation structure and its dynamic changes play a decisive role in the performance of aqueous batteries (Fig. 7d) [94]. Li *et al*. found that under the condition of negative electrode polarization, the interfacial water in the water-in-salt electrolyte exhibits a structure with the ‘H-up’ and blue shift of the O-H stretching frequency. This structural change significantly influences the performance and stability of the battery (Fig. 7e) [95]. Yang *et al*. utilized interfacial water as a spacer to prevent the agglomeration of graphene sheets. The interfacial water provides a strong repulsive force, which balances with the π-π bonds between the graphene sheets to avoid their agglomeration, thereby preparing multilayer graphene films (Fig. 7f) [96].

### Biology process

**Figure 8.**
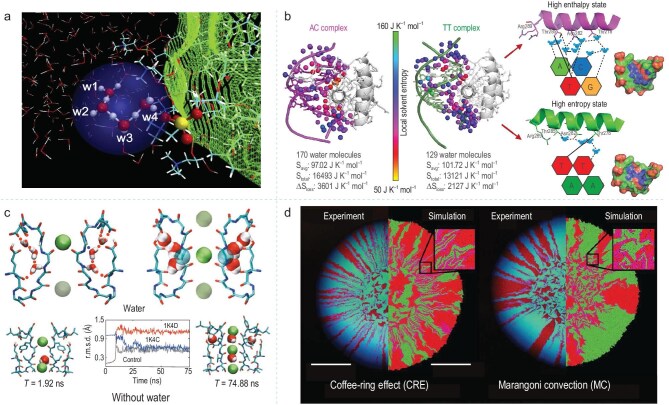
(a) Concerted proton transfer from the interfacial water to a phosphate group. Adapted with permission from [97]. Copyright 2018, licensed under CC BY 4.0. (b) Molecular dynamics analysis of entropies of resident water molecules at the BARHL_2_-DNAAC and BARHL_2_-DNATT interface and the schematic representation of the two optimal sites representing high enthalpy and entropy optima. Adapted with permission from [98]. Copyright 2025, Springer Nature. (c) Molecular dynamics simulations reveal the mechanism of recovery from inactivation. Adapted with permission from [99]. Copyright 2013, Springer Nature. (d) Spatial patterns that formed during surface-associated growth after cell deposition under different evaporation-induced hydrodynamics. Adapted with permission from [100]. Copyright 2023, Springer Nature.

Interfacial water plays a pivotal role in various physiological processes, including substance transport and signal transduction within living organisms. By influencing the interactions among biomolecules, such as protein folding and nucleic acid hybridization, it ensures the functionality and structural stability of biological macromolecules thereby providing a guarantee for the orderly operation of normal life activities. Nguyen *et al*. discovered that in the process of proton transfer between two phosphatidylcholine bilayers, the excess proton interacts with interfacial water molecules and tends to be located at the water/membrane interface. The interfacial water molecules have a specific orientation relative to the lipid bilayers. This interfacial water-proton interaction helps explain the proton mobility as experimentally observed at the membrane interface. (Fig. 8a) [97]. Morgunova *et al*. discovered that the key to dinucleotide recognition relies on the interfacial water molecules between proteins and DNA. Their indirect interactions lead to an entropy-enthalpy trade-off, influencing the binding affinities of transcription factors to different sequences and gene expression (Fig. 8b) [98]. Ostmeyer *et al*. employed molecular dynamics simulations to reveal that in the KcsA K⁺ channel, water molecules are of paramount importance in the inactivation and recovery processes of the channel. By regulating the binding and release of these water molecules, the transition time of the channel from a non-conductive state to a conductive state can be significantly affected (Fig. 8c) [99]. Ruan *et al*. discovered that during the cell deposition process dominated by the coffee-ring effect, the kinetic behavior of interfacial water regulates the initial deposition morphology of bacteria. This increases the contact probability between donor and recipient bacteria during colonization, thereby promoting the transfer of resistant gene plasmids (Fig. 8d) [100].

### Hydrovoltaic technology

**Figure 9.**
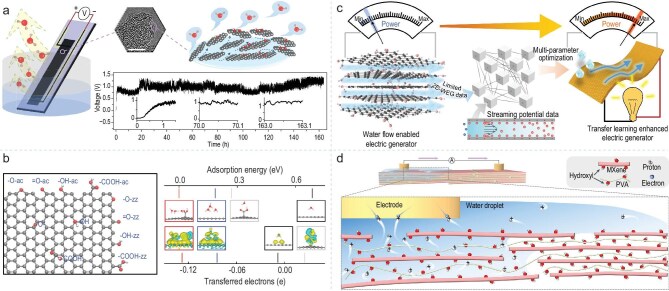
(a) Evaporation-induced electricity in CB. Adapted with permission from [101]. Copyright 2017, Springer Nature. (b) Schematic structure of a graphene flake with different functional groups, the synergistic effect of oxygen functional groups and protonation in tuning charge exchange between water and graphene. Adapted with permission from [102]. Copyright 2021, American Chemical Society. (c) TL enhanced 2D-WEG with integrated 2D channels. Adapted with permission from [103]. Copyright 2022, Springer Nature. (d) Schematic of the proposed mechanism for electricity generation in the MPCF. Adapted with permission from [104]. Copyright 2024, Springer Nature.

Interfacial water plays an indispensable role in the development of hydrovoltaic technology. It dynamically regulates the dynamics of ions on the surface of hydrovoltaic materials, establishing an asymmetric ion concentration gradient. This gradient drives the charge separation and directional migration within the interfacial region, thereby generating an electric potential difference. Figure 9a presents a typical water-evaporation-induced electricity device, which consists of a porous carbon black (CB) film with two carbon nanotube electrodes separately deposited at its two ends. Under ambient conditions, the device's bottom is immersed in water, which then rises within the CB film via capillary force. The generation of electrical energy is due to the interactions between interfacial water flow and the carbon film which is driven by evaporation. If the water supply is maintained, the electrical output can last for more than 150 hours [101].

Based on ab *initio* calculations, Xu *et al*. discovered that oxygen-containing groups such as hydroxyl and carboxyl groups in graphene oxide significantly enhance the binding energy and charge transfer efficiency between interfacial water and graphene oxide. In particular, the carboxyl groups exhibit a strong attractive force towards water molecules, promoting their protonation and strengthening the interaction with graphene. This provides an important theoretical basis for making full use of interfacial water to enhance the hydrovoltaic effect (Fig. 9b) [102]. Yang *et al*. developed a water-evaporation-induced electricity generator based on a graphene oxide film with 2D nanochannels. They utilized the adsorption and ionization of water on the surface of graphene oxide sheets to form an EDL. Subsequently, the directional flow of water promoted the charge separation within the EDL thus generating electrical energy. Meanwhile, by employing a transfer learning strategy to optimize the parameters related to interfacial water, they further enhanced the performance of hydrovoltaic devices (Fig. 9c) [103]. Xia *et al*. constructed a hydrovoltaic device based on an MXene/PVA composite film (MPCF) with a 2D nanochannel structure. The permeation of interfacial water within the nanochannels of the MPCF and dissociation of protons led to a high proton concentration inside the channel and the formation of counter-water-flow self-diffusion of protons inside the MPCF, thereby generating electrical energy (Fig. 9d) [104].

## SUMMARIES AND PROSPECTS

Research on interfacial water has broken the traditional approach of simplifying water as a background environment, as its significant roles in physical, chemical, and biological processes have increasingly come to light. Interfacial water exhibits numerous properties that are distinct from those of bulk water. These include physical properties such as molecular structure and arrangement order of water, electrical properties like dielectric constant and electrical conductivity, and hydrodynamic behaviors including surface tension and viscosity. These properties are of great significance for various fields, including hydrovoltaic effects, catalysis, biology, and the development of new materials. However, due to differences in theoretical calculation models, characterization techniques, and research materials, certain controversies persist in the current studies regarding the structure and dynamic behavior of interfacial water.

Future research directions that can be envisioned for interfacial water include the following: (1) explore the behavior of interfacial water under supercritical fluid conditions, ultra-low temperature environments, and within nano-confined spaces. By comprehensively understanding the microscopic interaction mechanisms of interfacial water under these special conditions, new research perspectives can be provided for the study of superconducting materials and the fabrication of quantum devices. (2) Combine multiple high-resolution characterization techniques to develop multi-dimensional and multi-scale precise characterization technologies. In combination with advanced computational simulation methods, new theoretical model systems should be developed to describe the multi-scale behaviors of these complex systems, thereby revealing the action mechanisms of interfacial water in extreme and complex environments. (3) Build a bridge between the basic scientific research and applications of interfacial water. Currently, the vast majority of research on interfacial water focuses on the discovery of its properties. Computer simulations and other means can be used to conduct extensive research under non-actual and even extreme conditions, which is divorced from the practical applications of interfacial water. Therefore, it is recommended to strengthen the connection between basic scientific research and practical applications to accelerate the use of interfacial water in fields such as the environment, materials, and energy.
